# Non-Syndromic Multiple Odontogenic Keratocyst: A Case Report

**Published:** 2013-09

**Authors:** N Kargahi, M Kalantari

**Affiliations:** a Dept. of Oral and Maxillofacial Pathology and Torabinejad Research Center, School of Dentistry, Isfahan University of Medical Sciences, Isfahan, Iran.; b Post Graduate, Dept. of Oral and Maxillofacial Pathology and Torabinejad Research Center, School of Dentistry, Isfahan University of Medical Sciences, Isfahan, Iran.

**Keywords:** Multiple Odontogenic Keratocyst, Gorlin-Goltz Syndrome, Non-Syndromic

## Abstract

Odontogenic keratocyst (OKC) is a common developmental odontogenic cyst affecting the maxillofacial region. Multiple OKCs are usually seen in association with nevoid basal cell carcinoma syndrome (NBCCS) but approximately 5% of patients with OKC have multiple cysts without concomitant syndromic presentation. This report represents a case of multiple OKCs in a non-syndromic patient

## Introduction

Odontogenic keratocyst (OKC) is a developmental odontogenic cyst with specific histopathologic features and clinical behavior. Several investigators suggested that OKCs must be regarded as a benign cystic neoplasm rather than a cyst.

In the latest WHO classification of odontogenic tumors in 2005, these lesions have been given the name “karatocystic odontogenic tumors” (KCOTs) [1]. 

Multiple KCOTs are usually seen with cutaneous, skeletal, ocular and neurologic abnormalities as a component of nevoid basal cell carcinoma syndrome (NBCCS). The features of this syndrome were first described by Gorlin and Goltz in 1960, so it is also recognized as Gorlin- Goltz syndrome [2].

KCOTs in the jaws arise from the cell rests of dental lamina and usually are seen during the second to fourth decades of life with a slight male predilection [3]. Typically, multiple KCOTs have been known to occur in association with NBCCS, but rarely may they be seen without concomitant syndromic manifestations [4].

This study reports a case of multiple KCOTs without any syndromic manifestations.

## Case report

An 11- year-old boy with a complaint of swelling in left side of the upper jaw was referred to our clinic. 

**Figure 1:**
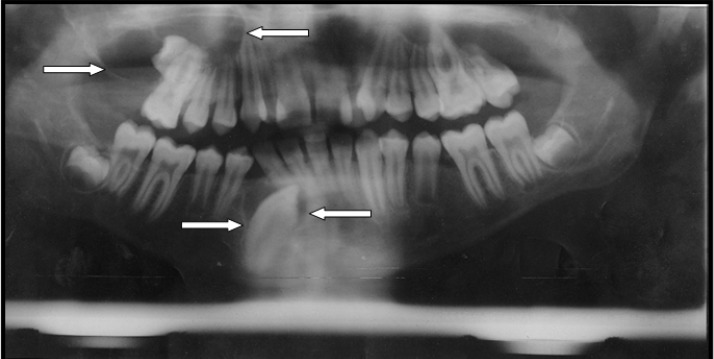
Panoramic radiograph showing two cyst-like radiolucencies in upper and lower jaws

Systemic signs and symptoms, past medical history and hematologic tests were within normal limits. The radiographies from chest and skull were unremarkable and no cutaneous abnormality was revealed. In panoramic radiograph, two radiolucencies with corticated border were revealed around the unerupted mandibular left canine and the unerupted maxillary left second molar area. Maxillary second molar was displaced (Figure1).

Regarding the radiographic examination and presence of unerupted teeth and their location, the initial differential diagnosis was dentigerous cyst and the second was KCOT. Other odontogenic cysts and tumors such as adenmatoid odontogenic tumor were considered as other differential diagnoses.

Enucleation of the cystic lesions was performed under local anesthesia and tissue samples were obtained for histopathologic examination. The surgical specimens were sheet- like with cystic appearance. After processing, the tissue samples were sectioned and stained with hematoxylin and eosin (H&E). 

**Figure 2a:**
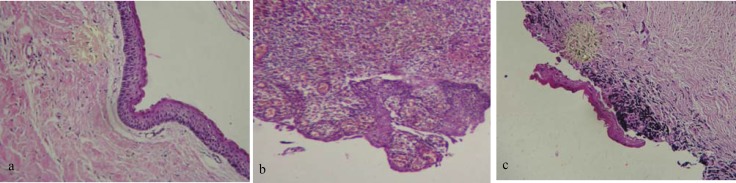
Mandibular lesion photomicrograph showing parakeratinized stratified squamous epithelium of uniform 5-6 cell thickness and palisaded basal cell layer without rete pegs (H &E Stain. 10x) **b ** Maxillary lesion photomicrograph showing an inflammatory cyst appearance with hyperplastic epithelial lining and rete ridge formation (H &E Stain. 10x) **c **Photomicrograph showing a small epithelial lining with KCOTs characteristics in the maxillary lesion. (H &E Stain. 10x

The histopathologic examination revealed that the cystic lining of mandibular lesion was corrugated parakeratinized epithelium with uniform thickness of 5-6 peg formation. The cyst wall was composed of a non-inflammatory fibrous connective tissue. All these features established the diagnosis of KCOT for the mandibular lesion (Figure 2a). But the maxillary lesion showed an inflammatory odontogenic cyst appearance with inflammatory cells infiltration in fibro-vascular connective tissue wall. The epithelial lining showed varying degrees of hyperplasia and rete ridge formation (Figure 2b).

With definitive diagnosis of KCOT in mandibular lesion and absence of any evidence of NBCCS in clinical examinations, multiple sections were cut from the maxillary lesion. These sections were obtained because of the probability of multiple non- syndromic KCOTs.

H & E slides were evaluated carefully and at last a small epithelial lining with characteristic features of KCOT was detected (Figure 2c).

According to these entire features which were correlated with the clinical and radiographic findings, the diagnosis of KCOT was established for both cystic lesions.

## Discussion

OKC is a common developmental odontogenic cyst and its biologic behavior is similar to a benign neoplasm [5]. Therefore, in the latest WHO classification of odontogenic tumors in 2005, it has been given the term keratocystic odontogenic tumor [1].

KCOT may be found in any age with peak prevalence between 10 to 40 years old [6].

The mandible is involved in 60 to 80% of cases with a marked tendency to occur in the posterior body and ascending ramus [5, 7].

Small KCOTs are usually asymptomatic but larger ones may show clinical manifestations like pain, swelling or drainage [7]. The presented case, revealed only the swelling.

Radiologically, KCOTs demonstrate a well defined radiolucent area with smooth and often corticated margins and may be unilocular or multilocular. In 25 to 40% of cases, an unerupted tooth is seen in association with the lesion [8]. Radiographic findings in mandibular lesion showed a unilocular radiolucency in relation with an unerupted canine. In maxilla, a multilocular radiolucent lesion in relation with an unerupted second molar was seen; which had well corticated margins.

In this case, microscopic evaluation of the mandibular lesion showed characteristic features of KCOT inducing a corrugated parakeratinized stratified squamous epithelial lining with palisaded basal cell layer without rete ridge formation [8].

Maxillary lesion showed histopathologic features of an inflammatory odontogenic cyst, but regarding the possibility of multiple KCOTs in this patient, several sections were prepared to achieve the correct diagnosis.

Finally we revealed characteristic features of KCOT in small area of histopathologic slides and the diagnosis of other cysts, especially inflammatory dentigerous cyst, was ruled out.

Finally, the diagnosis of KCOT was established for both cystic lesions.

Multiple KCOTs are usually considered as a component of Gorlin- Goltz syndrome or NBCCS [9], orofacial digital syndrome [10], Ehler- Danlos syndrome [11], Noonan syndrome [12] or other syndromes.

Rarely, multiple KCOTs are seen without other syndromic manifestations [4].

Brannon [6] reported that 5.8 percent of 312 cases of KCOTs, had multiple cysts without any syndromic manifestations. 

NBCCS is recognized by multiple KCOTs, nevoid basal cell carcinomas of the skin, bifid ribs, calcification of the falx cerebri and other features [2].

However, except for presence of KCOTs, our patient was healthy in clinical examinations and suggestive features of these syndromes such as basal cell carcinoma, skeletal or orofacial defects, stunted growth, bleeding diathesis, hyper-extensible skin and hypermobile joints and other features were not present. 

Multiple KCOTs might be the first and the only manifestation of NBCCS without any other features associated with syndrome. However, other symptoms can occur in later decades of life [13].

Similar cases to the current case have been reported in a few published English articles.

Auluck et al. [14] discussed a 22 year-old patient with multiple recurrent KCOTs in all four quadrants with a complaint of pus drainage over the previous week without pain or facial swelling. The patient had no any other features associated with NBCCS.

Sholapurkur et al. [3] presented a 24 year-old case with multiple non-syndromic KCOTs in both jaws with chief complaint of a slow growing swelling since 3 years and drainage since 15 days. The swelling was associated with pain with gradual onset radiating to head on same side. Lesions were cyst-like radiolucencies associated with impacted teeth on panoramic radiograph. 

Parikh [2] reported a 19- year-old case with two KCOTs in both jaws without any other concomitant syndromic features. The complaint was swelling for one year and pain for three months. Panoramic radiograph revealed two radiolucencies with corticated borders associated with impacted teeth.

Bartake et al. [4] reported a 20- year-old case with multiple recurrent KCOTs without any other noticeable features indicative of Gorlin syndrome. No recurrence occurred after 3- year follow up.

Guruprasad et al. [15] discussed a 16- year-old patient with multiple KCOTs and a complaint of slow progressing swelling in both jaws without any other features of syndrome.

Also, findings of Habibi et al. [16] study on Iranian populations showed that 8.1% of 83 cases with KCOTs, were associated with NBCCS and 7.6% of them had recurrence, but none of the cases with multiple KCOTs were non-syndromic. 

Therapeutic interventions of KCOT include marsupialization and enucleation, combined with adjuvant cryotherapy with Carnoy’s solution, and marginal or radical resection [16].

For unerupted permanent teeth in children, conservative treatment should be done first, because an aggressive operation can cause adverse effects on teeth development and its eruption. Marsupialization followed by enucleation has the lowest recurrence rate among the conservative treatments [2].

KCOTs related to NBCCS have more aggressive behavior and higher recurrence rates than non- syndromic ones.

This characteristic is due to a high proliferation rate of the epithelial linings in the syndromic cases [17].

There is a little information about the relationship between cell proliferative markers and the recurrence rate of KCOTs. The study performed by kuroyanagi et al. [18] showed that ki- 67 expression, at time of diagnosis, may act as a prognostic marker. In order to prevent the recurrence of the tumor, ki- 67 labeling index consideration can be helpful in adjunctive surgical procedures. 

Since our patient lived in a small village, following up and checking the recurrence of the lesions was impossible.

## Conclusion

In any patient with a KCOT, the presence of multiple KCOTs should be considered. Therefore, careful histopathologic examination for any other existing lesion should be done. Moreover, a complete clinical examination and long- term follow up must be performed to detect any other features associated with NBCCS.
